# Molecular and Evolutionary Basis of Avian Plumage Colour

**DOI:** 10.3390/biology15141178

**Published:** 2026-07-17

**Authors:** Lu Bai, Jun Yin

**Affiliations:** College of Life Science, Inner Mongolia Agricultural University, Hohhot 010018, China; 18847161374@163.com

**Keywords:** avian plumage, sexual dichromatism, colour evolution, melanin, carotenoids

## Abstract

The rapid growth of avian genomic resources has created a field rich in genomic data but still in need of integrative synthesis. Here, we review evidence linking four colour-producing systems—melanin, carotenoids, psittacofulvins and structural colouration—to feather development and evolutionary change. Melanin output is shaped by the *MC1R*–*ASIP*–*MITF* regulatory axis, melanogenic enzymes and the local availability of substrates such as cysteine. Carotenoid colouration depends on dietary uptake, SCARB1-mediated transport, *BCO2*-regulated precursor availability and, in many red species, *CYP2J19*-mediated ketolation acting with *BDH1L*; however, *CYP2J19*-independent red routes demonstrate that similar colour phenotypes can evolve through different molecular solutions. Structural colours arise from feather nanostructures and may interact developmentally with pigment deposition, whereas psittacofulvins represent an independent pigment system in parrots. We also discuss sex-biased gene expression, feather-region specificity, environmental sensitivity and lineage-specific regulatory change. Overall, avian plumage colour evolution is shaped by conserved biochemical and developmental constraints, yet similar colour phenotypes can arise through different regulatory, metabolic or structural routes.

## Simple Summary

Bird plumage colours are among the most diverse visual traits in vertebrates and are shaped by both natural and sexual selection. This review explains how melanin, dietary carotenoids, parrot psittacofulvins and feather nanostructures generate visible colour and how these mechanisms are regulated across sex, body region and lineage. Rather than treating pigment systems as separate topics, we compare them within a shared developmental and evolutionary framework. Particular attention is given to the *MC1R*–*ASIP*–*MITF* axis in melanin production, the SCARB1–*BCO2*–*CYP2J19*/*BDH1L* pipeline in carotenoid colouration, and *CYP2J19*-independent routes to red plumage. Together, these examples show that bird plumage colours arise from different combinations of pigment chemistry, feather development, gene regulation and ecological context.

## 1. Introduction

### 1.1. Background and Significance

The rapid expansion of avian genomic resources has made plumage-colour research rich in candidate genes, regulatory regions and comparative datasets. Yet many findings remain separated by pigment type, species and method. The Bird 10,000 Genomes (B10K) Project illustrates this transition. Its family-level phase released genomes representing 92% of avian families (363 species across 218 taxonomic families), and the project has moved towards genus-level sampling across approximately 2250 avian genera [1]. Complementary initiatives, including the Vertebrate Genomes Project, further emphasise chromosome-level assemblies for phylogenetically diverse vertebrate and avian lineages [2]. Compared with 2010, when only three avian genomes (chicken, zebra finch and turkey) were available [3], the present genomic landscape creates a much stronger basis for comparative synthesis.

Several influential reviews have provided important foundations for this field. Hubbard et al. [4] surveyed vertebrate pigmentation before the current transcriptomic and epigenomic expansion; Roulin and Ducrest [5] reviewed avian colour genetics within a largely Mendelian framework; and more recent syntheses have addressed colour-evolution genomics, macroevolutionary diversification and emerging questions in avian colouration [6,7,8]. The remaining need is not to replace these contributions but to connect their insights more explicitly across molecular mechanisms, feather development, ecological context and macroevolutionary pattern.

Building on these studies, this review develops a mechanistic synthesis of how avian plumage colours are produced, regulated and diversified through interactions among pigment chemistry, feather nanostructure, gene regulation and ecological signalling. It compares melanin, carotenoid, psittacofulvin and structural colour systems within a shared developmental framework; highlights recent genomic and transcriptomic work on single-gene convergence, region-specific sexual dichromatism and epigenetic colour patterning [9,10,11]; and evaluates how pigmentation genetics may affect the reliability of plumage signals under environmental change. The review therefore emphasises feather-based colour traits and refers to non-feather integumentary colouration, such as beak or bare-skin colouration, only where it clarifies shared pigment-processing mechanisms.

Research on avian plumage has moved through three broad phases. The natural-history phase, shaped by Darwin and Wallace, established sexual selection, camouflage and adaptive colouration as central evolutionary problems [12,13]. The chemical phase, advanced by investigators such as Otto Völker, used chromatographic methods to identify major pigment classes, including melanins and carotenoids. The current genomic phase now makes it possible to connect these pigment classes to regulatory genes, feather development and evolutionary diversification at single-gene and genome-wide scales [14].

Plumage colours contribute to species recognition, mate choice, camouflage, thermoregulation and sexual selection [14]. Feathers are keratinised epidermal organs with hierarchical structures spanning molecular, cellular, tissue and whole-feather scales; this architecture allows pigment deposition and optical nanostructure to generate colour patterns more diverse than those of mammalian pelage or reptilian scales [15]. Fossil evidence indicates that melanin-based colouration has deep evolutionary roots, whereas carotenoid-based plumage appears to have diversified more recently, with many origins reconstructed after the Miocene in passerines and earlier origins inferred in some lineages [2,16]. Despite substantial progress, four questions remain especially important. First, the regulatory networks that generate sex-specific and feather-region-specific expression of pigmentation genes are still incompletely known. Second, the routes to red carotenoid plumage are more diverse than once assumed: many lineages use *CYP2J19*-mediated ketolation, whereas house finches (*Haemorhous mexicanus*) achieve red plumage without relying on this canonical route [17,18,19], and other lineages have lost functional *CYP2J19* without replacement [20]. Third, pigment deposition and feather morphogenesis interact during pattern formation, but the molecular mechanisms linking these processes remain only partly resolved. Fourth, the costs and reliability of carotenoid-based signals under dietary and climatic variation need to be tested quantitatively at molecular and population scales.

**Figure 1:**
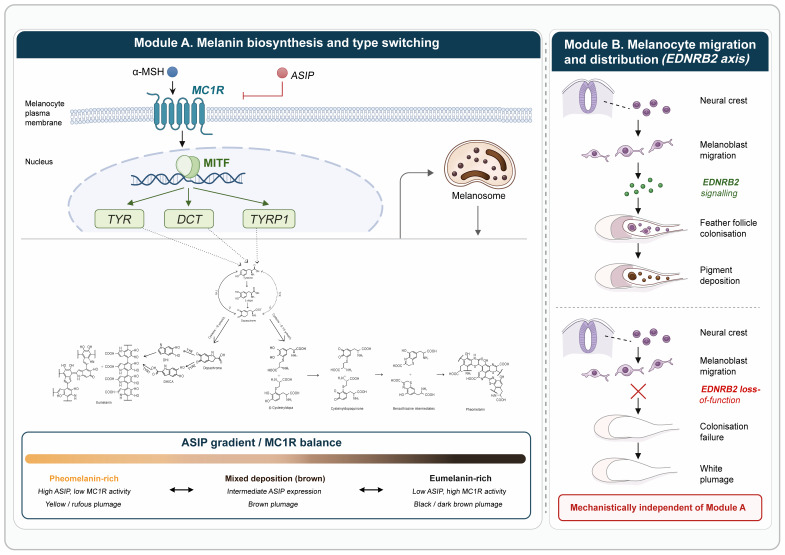
Integrated regulatory architecture of melanin-based plumage colouration in avian feather follicle melanocytes.

**Figure 2:**
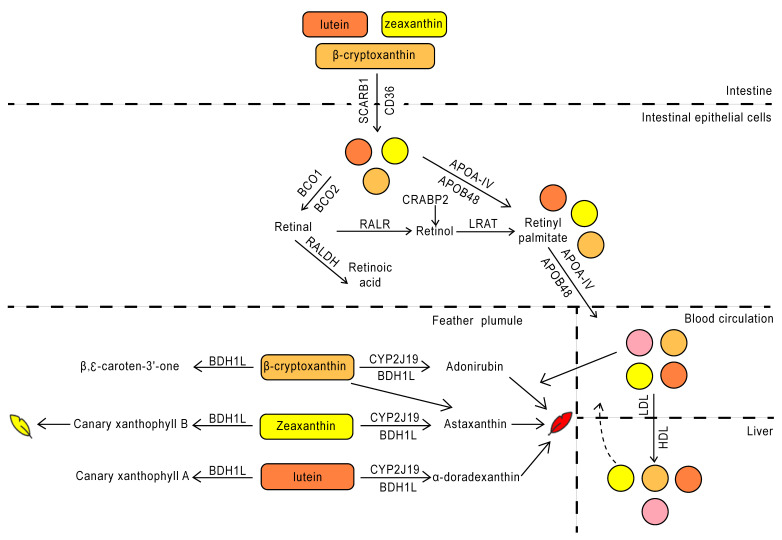
Complete carotenoid processing pathway from dietary intake to feather pigmentation.

**Figure 3:**
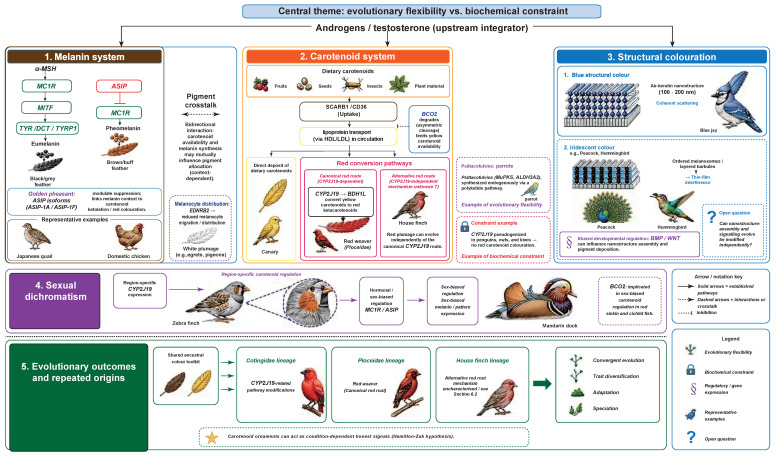
Schematic overview of major plumage colour-production pathways and their genetic regulation in birds.

These questions point to a central tension in avian plumage evolution: similar colour phenotypes may arise through different molecular routes, yet those routes are constrained by available enzymes, transport systems, feather developmental programmes and receiver perception. Routes to red carotenoid plumage illustrate this tension particularly clearly: red plumage has evolved repeatedly but only within a limited set of chemically feasible solutions. Melanin-based depigmentation provides a parallel case, because white plumage can arise through several distinct molecular routes affecting melanocyte migration, regulatory architecture or pigment synthesis. We use these paired examples to examine evolutionary flexibility within biochemical and developmental constraints, while treating melanin, carotenoid, psittacofulvin and structural colour systems within a single framework rather than as isolated topics. The schematic figures guide this synthesis: Figure 1 and Figure 2 summarise melanin regulation and carotenoid processing in greater detail, whereas Figure 3 integrates the major colour-production pathways and their regulatory connections.

### 1.2. Mechanism Classification of Feather Colouring

The colour of avian plumage is produced by two major mechanisms: pigment colouration and structural colouration. Pigmentary and structural colours differ in their proximate bases: pigmentary colours depend on selective absorption by chemical pigment molecules, mainly melanins and carotenoids, whereas structural colours arise from physical interactions between light and feather microstructures [21]. Colour polymorphism in birds fulfils multiple functions, including camouflage, sexual selection and temperature regulation, with both genetic and environmental factors contributing to phenotypic variation [12,13]. These complementary mechanisms are fundamental for explaining the full range of avian plumage colour diversity. The mechanisms by which colour is produced, however, cannot be fully evaluated without considering how that colour is perceived—a dimension addressed in Section 1.3.

### 1.3. Colour Perception and Adaptive Signalling

Colour in birds is not only a product of pigment chemistry or feather optics; it is also a signal filtered by the visual system of the receiver [22,23]. Birds typically possess four classes of retinal cone photoreceptors: long-wavelength-sensitive (LWS, λmax~565 nm), medium-wavelength-sensitive (MWS, ~505 nm), short-wavelength-sensitive type 2 (SWS2, ~455 nm), and a short-wavelength-sensitive type 1 class (SWS1) tuned either to ultraviolet wavelengths in UVS species (λmax~355–380 nm) or violet wavelengths in VS species (λmax~400–425 nm). Coloured oil droplets in inner cone segments act as spectral filters, narrowing photoreceptor sensitivities and improving chromatic discrimination.

This tetrachromatic system creates ultraviolet and violet signalling channels that may be highly salient to conspecifics but inconspicuous to humans or many mammalian predators. As a result, the adaptive significance of plumage colours depends on how they are detected and discriminated in avian colour space, not merely on how pigments or nanostructures generate reflectance spectra [24,25].

Colour production and perception are embedded within the hierarchical feather architecture, which simultaneously supports flight, thermoregulation, waterproofing and visual signalling [26,27]. Pigments are localised within structurally defined feather regions rather than deposited uniformly. For this reason, we treat feather morphogenesis, pigment deposition and signal perception as connected components of plumage-colour evolution rather than as separate levels of analysis.

## 2. Major Pigment Types and Biosynthetic Pathways

### 2.1. Melanin

#### 2.1.1. Biochemical Branch Point and Substrate Control

Melanocytes originate from the neural crest and migrate into the skin and feather-forming regions during embryonic development [28]. Within feather follicle melanocytes, tyrosinase (*TYR*) converts tyrosine to dopaquinone (DQ), the common precursor of eumelanin and pheomelanin. DQ is the major biochemical branch point in melanin synthesis. When cysteine is abundant, DQ couples with cysteine to form cysteinyldopa intermediates that are subsequently oxidised and polymerised into pheomelanin.

When cysteine is limited, DQ instead undergoes cyclisation to dopachrome. Dopachrome is then channelled either through *DCT*/*TYRP2* to 5,6-dihydroxyindole-2-carboxylic acid (DHICA) or through spontaneous decarboxylation to 5,6-dihydroxyindole (DHI), and these products are polymerised into eumelanin with the involvement of *TYRP1* [29]. In vitro threshold values for cysteine and cysteinyldopa support this substrate-competition model [30], but these values should be interpreted as context-dependent properties of melanocytes rather than fixed constants. Together, these reactions place cysteine availability at the dopaquinone branch point: higher cysteine availability favours cysteinyldopa formation and pheomelanin synthesis, whereas lower cysteine availability favours dopachrome formation and eumelanin synthesis.

Cysteine availability is controlled by transport, metabolic allocation and nutritional state. Transporters such as *SLC7A11* and *CTNS* influence the intracellular cystine/cysteine pool [31,32,33], whereas metabolic partitioning allocates cysteine among glutathione synthesis, redox balance, degradation and pheomelanin production [34]. Environmental and nutritional inputs may also enter this layer: food availability has been associated with *CTNS* expression and cysteine homeostasis in gyrfalcons, and zebra finch studies link pheomelanin production with cysteine detoxification [33,34,35]. These findings indicate that cysteine is not simply a passive substrate but a regulated supply layer that interacts with *MC1R*–*ASIP* signalling and feather-follicle context to shape melanin output.

The expression of melanogenic enzymes is coordinated by microphthalmia-associated transcription factor (*MITF*), whose activity is influenced by the *MC1R*–*α-MSH* pathway described below. Figure 1 summarises these relationships by separating melanin-type switching within feather follicle melanocytes from melanocyte spatial distribution during feather development.

**Module A** shows melanin-type switching within feather follicle melanocytes. α-MSH activates *MC1R* and promotes *MITF*-mediated expression of *TYR*, *DCT* and *TYRP1*, whereas *ASIP* antagonises *MC1R* signalling. Cysteine availability is positioned at the dopaquinone branch point because it biases output towards pheomelanin or eumelanin. **Module B** shows *EDNRB2*-dependent melanocyte migration and follicle colonisation; disruption of this distribution process can produce white or piebald plumage independently of pigment synthesis. Solid arrows indicate direct regulatory or biosynthetic relationships, blunted lines denote antagonism, and dashed arrows indicate transcriptional regulation.

#### 2.1.2. MC1R–ASIP–MITF Signalling and Melanin-Type Switching

##### Melanocortin 1 Receptor (MC1R)

Classical breeding studies in domestic chickens (*Gallus gallus domesticus*) and Japanese quail (*Coturnix japonica*) identified the Extension and Agouti loci as major determinants of melanin-type switching before their molecular identities were known [36,37]. The subsequent cloning of *MC1R* as the Extension locus and *ASIP* as the Agouti locus confirmed that these loci regulate the balance between eumelanin and pheomelanin deposition [5,36]. This historical sequence is important because it shows that the core functional logic of the *MC1R*–*ASIP* axis was already recoverable from phenotype-to-genotype inference.

*MC1R* encodes a seven-transmembrane G protein-coupled receptor that promotes eumelanin synthesis when activated [36,38]. Its principal endogenous agonist is α-melanocyte-stimulating hormone (α-MSH), a peptide derived from pro-opiomelanocortin (*POMC*) and released within the feather follicle microenvironment [29]. Binding of α-MSH to *MC1R* activates downstream signalling that enhances *MITF*-driven expression of melanogenic enzymes; when *MC1R* is not stimulated, or when it is antagonised by *ASIP*, the pathway shifts towards pheomelanin production.

Functional evidence from quail supports this model. *MC1R* expression is higher in black quail than in white or wild-type quail, and transient *MC1R* expression in wild-type quail embryos increases skin melanin deposition [37]. *MC1R* orthologues are highly conserved across vertebrates [39], consistent with the ancient origin and broad functional importance of this receptor in melanin-based colouration.

##### Agouti Signalling Protein (ASIP)

*ASIP* encodes a secreted antagonist of *MC1R* that competitively displaces α-MSH, suppresses eumelanin synthesis and favours pheomelanin production [5]. Its effect is gradient-dependent: low or absent *ASIP* expression sustains *MC1R* activation and black eumelanin deposition; intermediate expression produces mixed eumelanin–pheomelanin phenotypes; and high expression biases the pathway towards pheomelanin. Functional assays in Japanese quail and spatiotemporal expression studies in chickens support this regulatory relationship [40,41].

In the golden pheasant (*Chrysolophus pictus*), *ASIP* alternative splicing elaborates *MC1R*–*ASIP*-mediated melanin-type regulation. *ASIP-1A* and *ASIP-1F* differ in their effects on *MC1R* antagonism and eumelanin suppression, generating pheomelanin-biased melanin backgrounds. A red or orange hue in mixed-colour plumage can also involve carotenoid components: where ketocarotenoids contribute to the final colour, *CYP2J19* acts within carotenoid ketolation, while *ASIP* splice forms shape the melanin background. *MITF-1M* isoforms enriched in pheomelanin feathers may coordinate downstream enzyme expression [42]. Thus, the golden pheasant example refines the *MC1R*–*ASIP* model, whereas *EDNRB2*-related cases represent melanocyte distribution and depigmentation.

#### 2.1.3. Melanocyte Distribution and Plumage Depigmentation

##### Endothelin Receptor B2 (EDNRB2)

Beyond *MC1R* and *ASIP*, which regulate melanin synthesis and type switching, *EDNRB2* controls melanocyte spatial distribution. *EDNRB2* encodes an endothelin receptor required for melanoblast migration during embryonic development; loss-of-function mutations can prevent melanocyte colonisation of feather follicles and thereby produce white or piebald plumage [43]. Domestic geese provide several examples of this distribution-level mechanism. In Chinese domestic swan geese (*Anser cygnoides domesticus*), a 14 bp frameshift insertion in *EDNRB2* is associated with white plumage, and comparative genomic work indicates that distinct *EDNRB2* variants have segregated independently in graylag and swan geese [44,45]. A codominant *EDNRB2* allele also controls white breast patch patterning in Wugang Tong geese [46].

Domestic pigeons (*Columba livia*) provide a parallel case. A missense mutation (p.Val295Met) identified by population genomic screening causes recessive white plumage, and an allelic series at *EDNRB2* generates patterns ranging from complete white to intricate pied phenotypes [47,48]. Together, the goose and pigeon cases highlight the separation between pigment-cell distribution and pigment synthesis: *MC1R*–*ASIP* changes the melanin type within melanocytes, whereas *EDNRB2* changes whether melanocytes successfully colonise the feather follicle.

##### Additional Mechanisms of Plumage Depigmentation

Beyond these distribution-level cases, white plumage can also arise from disruptions at other levels of the pigmentation hierarchy, including retinoid-associated signalling (*RAI14*), master-regulator architecture (*MITF*), and pigment-synthesis enzymes such as tyrosinase. The following examples illustrate these additional routes.

In chickens, a genome-wide association study integrating a 600K SNP panel identified *RAI14* (retinoic acid-induced protein 14), a Z-chromosome-linked gene, as a candidate regulator of plumage pigmentation; indel variants within *RAI14* are significantly associated with white versus coloured plumage phenotypes, and *RAI14* mRNA is expressed across all tissues examined, including the feather bulb [49]. Unlike the retinoid metabolic intermediates discussed in Section 2.2 in the context of carotenoid processing, *RAI14* functions within the retinoic acid signalling pathway—acting downstream of nuclear retinoic acid receptors to regulate melanocyte differentiation programmes—rather than in pigment biosynthesis per se. This represents a third class of depigmentation mechanism, distinct from both synthesis switching (*MC1R*–*ASIP*) and migration failure (*EDNRB2*).

Given *MITF*’s established role as the master transcriptional coordinator of melanin synthesis genes (Section 2.1 and Figure 1), it is notable that structural variation within *MITF* itself—rather than in its downstream targets—can also produce depigmentation. In ducks, pan-genome analysis has revealed that a 6634 bp Gypsy transposon insertion in an *MITF* intron generates a novel transcript isoform that disrupts normal *MITF* function, contributing to white plumage formation during domestication [50]. This finding demonstrates that genomic structural variation in a master regulatory gene, mediated by transposable element activity, constitutes a fourth mechanistic route to white plumage—one that operates at the transcriptional architecture level rather than at the level of individual pigmentation enzyme genes.

Together, these cases show that white plumage is a convergent phenotype with a heterogeneous molecular aetiology. The same visible outcome can result from distribution failure, regulatory disruption, structural variation in a master regulator, or loss of pigment-synthesis capacity. This diversity supports the modular view developed in this review: similar plumage phenotypes can be produced by perturbing different levels of the pigmentation hierarchy.

#### 2.1.4. Melanosome Structure and Colour-Pattern Formation

Melanosomes are pigment-containing organelles whose size, shape, density and intracellular arrangement influence the final plumage phenotype. Their physical properties are developmentally regulated outputs, not passive containers of melanin. At the cellular level, *MITF*-driven expression of *TYR*, *DCT* and *TYRP1* controls pigment synthesis, whereas melanosome morphology and distribution help determine how that pigment is presented within feather tissue [29,51].

Melanin-based colouration also has functions beyond sexual signalling, including protection against structural damage, abrasion and ultraviolet radiation [4,52]. At larger scales, pigment pattern formation depends on follicle cycling, melanocyte stem-cell niche topology and signalling interactions between melanocytes and keratinocytes. Periodic patterns such as bands and bars can emerge from activator-inhibitor dynamics during follicle cycling, with pattern wavelength shaped by the kinetics of cell–cell signalling [53]. The spatial arrangement of melanocyte progenitors within the follicle niche further constrains the complexity of the resulting pattern [54,55,56].

At the molecular level, a cis-acting mutation at the *GJA5* locus mediates the within-feather Melanotic pattern in chickens, while autosomal barring is strongly associated with *MC1R* variation [57,58]. More recent work implicates epigenetic regulation in coupling tissue growth with periodic colour patterning [11], and candidate-locus reviews on ducks identify genes spanning melanin synthesis, carotenoid metabolism and structural colouration [59]. These findings show that colour patterning is encoded at cellular, tissue and genomic levels simultaneously.

### 2.2. Carotenoids

Carotenoids produce many yellow, orange and red colours in vertebrate integuments and often contribute to sexually selected ornaments [4]. Unlike melanin, carotenoids are not synthesised de novo by birds and must be acquired from the diet; their expression is therefore strongly influenced by environmental availability and physiological allocation [60]. Avian carotenoids include oxygenated xanthophylls, such as lutein, zeaxanthin, antheraxanthin and astaxanthin, and non-oxygenated carotenes, such as β-carotene and lycopene.

Chemically, carotenoids are tetraterpenoid pigments built from eight isoprene units and characterised by an extended conjugated double-bond system. Their colour depends on both molecular structure and optical context. In birds, the visible outcome is further shaped by uptake, transport, enzymatic conversion, tissue-specific deposition and interaction with feather proteins or nanostructures [61]. For example, astaxanthin contributes to the pink flush of Franklin’s and ring-billed gulls (*Larus pipixcan* and *L. delawarensis*) [62], while comparative work on other vertebrates reminds us that carotenoid-derived colours often interact with other pigmentary or structural components [63].

#### 2.2.1. Carotenoid Uptake, Cleavage, Transport and Feather Deposition

##### Intestinal Absorption and Entry into the Systemic Pool

Birds acquire carotenoids from the diet and either deposit them directly into integumentary tissues or enzymatically modify them before deposition. The first control point is intestinal uptake. Bile-solubilised carotenoids are absorbed by enterocytes primarily through SCARB1 and CD36, with NPC1L1 contributing to cholesterol–carotenoid co-transport [64,65,66,67]. Comparative transport studies support a conserved lipid-uptake logic: SCARB1 mediates cellular carotenoid uptake in Drosophila, and a CD36-related protein cooperates with lipid-binding proteins during selective carotenoid transport in silkworms [68,69].

In birds, the relevance of this transport step is clearest for SCARB1. Loss of SCARB1 function disrupts carotenoid uptake and can abolish carotenoid-based colouration [70], and comparative work supports functional conservation of SCARB1 across vertebrates [71]. Selective absorption of xanthophylls such as lutein may depend partly on transporter substrate specificity [72], while species-specific preferences for carotenoid classes can be reflected in plumage colour [73]. Carotenoids may also be stored in fat, suggesting that adipose tissue can act as an overlooked reservoir for later feather deposition [74]. Figure 2 summarises this gut-to-feather pipeline.

##### Carotenoid Cleavage and Retinoid Handling

After uptake, some carotenoids enter retinoid metabolism. Cytosolic β-carotene-15,15′-monooxygenase 1 (*BCO1*) symmetrically cleaves provitamin A carotenoids such as β-carotene to generate retinal [75]. Retinoid handling is influenced by intracellular binding and processing proteins, including CRABP2, which has been characterised in retinal pigment epithelial systems [76]. Retinal can be reduced to retinol and subsequently esterified by retinol acyltransferases, including *LRAT*, to form retinyl esters [77]. This pathway connects dietary carotenoids with vitamin-A metabolism, visual physiology and broader retinoid signalling.

By contrast, mitochondrial β-carotene-9′,10′-oxygenase 2 (*BCO2*) mediates asymmetric cleavage of carotenoids, including xanthophylls such as lutein, zeaxanthin and β-cryptoxanthin, producing apo-carotenoid products [68,71,78,79]. Because *BCO2* reduces the carotenoid pool available for deposition, reduced *BCO2* activity can promote tissue-specific carotenoid accumulation [80]. In avian plumage, *BCO2* is therefore best understood as a quantitative regulator of precursor availability: it influences how much yellow carotenoid remains available for direct deposition or further enzymatic modification. Coding variation in *BCO2* associated with carotenoid concentration in Chinese chicken skin further supports this interpretation [81,82]. The regulatory mechanisms controlling *BCO2* expression remain incompletely resolved; its tissue-specific and sex-biased regulation is considered further in Section 2.2.3 and Section 3.1.2.

##### Lipoprotein-Mediated Transport and Feather-Follicle Deposition

Once absorbed, carotenoids are packaged into lipoprotein particles and transported through the circulation. Chylomicron assembly depends on MTP and ApoB48, while ApoA-IV and *LCAT* contribute to particle maturation [83,84]. Lipoprotein lipase (LPL) hydrolyses circulating particles, and hepatic processing through LDL/HDL-associated pathways redistributes carotenoids to peripheral tissues [85]. These lipid-transport steps matter because carotenoids are hydrophobic molecules: their movement from gut to liver, blood and feather follicles depends on the same physiological infrastructure that handles neutral lipids and retinoids.

In birds, carotenoids ultimately reach developing feather follicles, where lipid droplet-mediated transport and local metabolism contribute to pigment deposition; songbirds also show a capacity for local carotenoid modification in the skin [86]. What remains poorly resolved is flux: real-time movement of carotenoids from gut absorption through hepatic processing to feather deposition has rarely been measured in living birds. Stable-isotope tracing and metabolomics across moult stages would help determine whether individual colour variation reflects uptake efficiency, hepatic conversion or follicle-level deposition.

The upper panel shows intestinal uptake through SCARB1/CD36 and the cleavage of carotenoids by *BCO1* and *BCO2*. The circulation panel shows lipoprotein-mediated transport through chylomicron, LDL and HDL pathways. The feather-follicle panel shows direct deposition of yellow carotenoids and enzymatic conversion of yellow precursors to red ketocarotenoids through the canonical *CYP2J19*–*BDH1L* route.

#### 2.2.2. Carotenoids in Avian Plumage Colouration

Carotenoid deposition in feathers depends on both systemic supply and local follicle biology. In birds, carotenoids enter developing feather follicles through lipid-associated transport and then bind to feather keratin or other proteins to form pigment deposits. Carotenoid deficiency disrupts normal plumage colouration; for example, canaries (*Serinus canaria*) that ingest insufficient carotenoids during moulting exhibit lighter plumage and develop white feathers [21,87]. A genomic survey identified 48 candidate genes linked directly or indirectly to lipid metabolism and carotenoid-based colour variation [42]. These candidates show tissue-specific expression across carotenoid-containing plumage and bill tissues, indicating that colour output depends on where and when lipid-transport and pigment-processing genes are active [88]. Within this gene set, SCARB1 provides a mechanistically clear example because it links systemic lipid transport to visible plumage colour: as a receptor for HDL and related lipid particles, its activity influences how much dietary pigment reaches feather follicles [70].

Analytical methods also shape our understanding of this pathway. Raman spectroscopy provides a non-destructive approach for detecting carotenoids in feathers [89], complementing HPLC and spectrometric methods that distinguish pigment composition across red, orange and yellow plumage [90,91,92,93]. For example, analyses of golden pheasant feathers have shown that red, orange and yellow regions contain carotenoids, but pigment identity alone does not fully explain final hue; gene expression, enzymatic modification and feather optical context must also be considered [89,91,92,93]. These techniques are therefore most informative when interpreted alongside gene-expression and metabolic data rather than as purely descriptive pigment surveys.

#### 2.2.3. Metabolic Modifications

Although birds cannot synthesise the carotenoid carbon skeleton de novo, many species modify ingested precursors into pigments not present in the diet. The final ornamental molecules deposited in feathers may therefore differ from the dietary molecules from which they originated. Two broad modification routes are important. Dehydration of dietary xanthophylls such as lutein can produce canary-type yellow pigments, including anhydrolutein and dehydrolutein. Oxidative modification of yellow precursors such as zeaxanthin and β-cryptoxanthin generates red ketocarotenoids, including astaxanthin and canthaxanthin [18]. In birds, *CYP2J19* is the principal cytochrome P450 ketolase implicated in this canonical red route, and the resulting ketocarotenoids contribute to red feathers, bare skin and beaks [19,94].

##### Red Colouration Genetics

Testosterone can upregulate *CYP2J19* expression through androgen-responsive elements in hepatic tissue and feather follicles, linking reproductive physiology with ketocarotenoid production and deposition [95]. *CYP2J19* has also been implicated in retinal carotenoid metabolism, connecting pigment modification with aspects of avian visual physiology. *BDH1L* acts with *CYP2J19* in the conversion of yellow carotenoids to red ketocarotenoids [96], and *CYP2J19* expression is associated with red colouration in weaverbirds [97]. In mixed-colour phenotypes such as golden pheasant plumage, *CYP2J19* contributes to carotenoid ketolation, whereas *ASIP* splice forms mainly shape the melanin background [42]. These findings support a canonical red route in which endocrine state, enzymatic capacity and feather-region expression are integrated.

In the zebra finch, genetic mapping first linked *CYP2J19* to a red feather colour [98]. *CYP2J19*-mediated red colouration has also evolved independently in other avian lineages [19]. This repeated recruitment is consistent with overlapping selective pressures, including sexual selection for red ornaments, but convergence in phenotype does not by itself prove identical selective agents in every lineage. In *Ploceidae*, the *CYP2J19* expression level is positively correlated with red colour intensity [97], providing one example in which gene expression and phenotype covary across species.

Not all red plumage depends on *CYP2J19*. House finches (*Haemorhous mexicanus*) produce bright red feathers through a *CYP2J19*-independent route [17]; the responsible mechanism—whether a paralogous cytochrome P450 enzyme, another oxidative enzyme or a non-enzymatic route—remains to be functionally resolved (see Section 6.2). Other lineages, including penguins, owls and kiwis, have pseudogenised *CYP2J19* and lack red carotenoid plumage [20]. The comparison reveals both flexibility and constraint: birds can reach red through more than one route, but the available routes are still bounded by carotenoid chemistry and enzymatic feasibility.

##### Yellow Colouration Genetics

Yellow colouration usually reflects direct deposition of unmodified dietary carotenoids, such as lutein and zeaxanthin, into feather tissue without ketolation [99]. In domestic chickens, yellow skin reflects a hybrid origin involving introgression from the Asian grey jungle fowl [100]. In the Northern Flicker (*Colaptes auratus*), yellow–red differentiation is associated with the distribution of specific carotenoid pigments [101]. These examples distinguish direct deposition of yellow precursors from enzymatic conversion to red ketocarotenoids.

As introduced in Section 2.2.1, *BCO2* regulates the carotenoid pool through asymmetric cleavage rather than acting as a colour-producing ketolase. Its relevance to yellow and orange traits is therefore quantitative: reduced *BCO2* activity can allow carotenoids to accumulate, whereas higher activity can deplete substrates before deposition [71]. Darwin’s finches illustrate this role because *BCO2* polymorphism is closely associated with beak-colour variation, and loss-of-function variants lead to carotenoid over-accumulation and brighter orange or yellow beaks [102]. In vitro work with purified chicken *BCO2* has clarified substrate specificity [103], while comparable associations between *BCO2* variation and carotenoid accumulation in cattle and sheep support a conserved vertebrate role [104,105]. In birds, the visible outcome still depends on how cleavage interacts with transport, deposition and feather-region-specific regulation; its possible contribution to sex-biased carotenoid retention is discussed in Section 3.1.2.

##### Other Metabolic Genes

Retinol isomerase (*RPE65*) is involved in retinol metabolism and is primarily associated with visual function [106]. It illustrates how carotenoid-derived compounds can be partitioned among colouration, vision and broader retinoid physiology.

#### 2.2.4. Psittacofulvins: Endogenous Parrot Pigments

Parrots provide an important case outside the dietary carotenoid framework because their red and yellow feathers are produced by psittacofulvins, a structurally and biosynthetically distinct pigment class.

Psittacofulvins are synthesised endogenously through a psittacine-specific polyketide pathway. A polyketide synthase, *MuPKS*, is required for pigment production, and loss-of-function mutations abolish yellow pigmentation in domesticated budgerigar mutants [107,108]. *ALDH3A2* modifies psittacofulvin aldehydes into carboxylic forms, and the ratio of aldehydic to carboxylic psittacofulvins contributes to red, orange, yellow and green feather phenotypes. In budgerigars (*Melopsittacus undulatus*), yellow psittacofulvins combine with a blue structural colour to generate green plumage [108]. This pathway shows that similar visible colours can evolve from pigment systems that are chemically and genetically distinct from carotenoids. Examples outside birds further illustrate that pigmentation pathways can evolve through unusual biochemical routes [109].

Because psittacofulvins are produced endogenously, their ecological interpretation differs from that of carotenoid-based colouration. In carotenoid systems, visible colour can reflect dietary access, intestinal uptake, systemic transport, enzymatic conversion and follicle deposition. In psittacofulvin systems, colour variation is more likely to depend on local pigment synthesis, chemical modification and feather-follicle regulation. Psittacofulvins are therefore best discussed as an endogenous parrot pigment system with its own biochemical and ecological implications.

Psittacofulvins also provide a useful link between pigment chemistry and feather optics. The budgerigar example shows how an endogenous yellow pigment can combine with a blue structural colour to produce green plumage. Similar visible colours therefore do not necessarily imply similar biochemical origins; in birds, red, yellow and green phenotypes can arise through different combinations of pigment chemistry, feather structure and gene regulation.

#### 2.2.5. Carotenoid Diversity and Genetic Complexity

At least 39 carotenoids have been identified in avian plumage, most derived from dietary precursors such as lutein, zeaxanthin, β-carotene and β-cryptoxanthin [42]. Variation in house finch plumage across age, subspecies and ornament colour illustrates how diet, metabolism, moult stage and regulatory context jointly shape carotenoid deposition [110].

Genetic knowledge alone is therefore insufficient to explain carotenoid colour variation. Although key enzymatic and transport genes such as *CYP2J19*, *BCO2* and SCARB1 have been identified, the regulatory architecture controlling carotenoid allocation across tissues, sexes and developmental stages remains incompletely characterised. Carotenoid metabolism requires uptake, transport, storage, enzymatic conversion and deposition; each step may contribute to individual or species-level differences in colour expression.

Carotenoid-based colour variation can be organised into three outcomes. Yellow colouration often reflects direct deposition of dietary xanthophylls. Canonical red colouration requires enzymatic conversion of yellow precursors, most prominently through the *CYP2J19*-*BDH1L* route. Alternative red routes, exemplified by house finches, show that equivalent visual phenotypes can emerge without the canonical ketolase. These outcomes represent different degrees or types of modification applied to the same dietary precursor pool, rather than three equivalent branches of a single pathway.

The diversity of carotenoid pigments across birds can be summarised by three linked parameters: the dietary precursors that are available and absorbed; the enzymatic capacity for *CYP2J19*/*BDH1L*-mediated ketolation or alternative red routes; and the spatial regulation of *BCO2*-dependent cleavage. Habitat, diet, hormonal state and feather-region-specific gene expression can each shift these parameters, linking carotenoid biochemistry to sexual dichromatism and evolutionary diversification.

Viewed as an integrated pathway, carotenoid colouration depends on serial changes in substrate availability. SCARB1 and CD36 influence the entry of dietary carotenoids into the systemic pool; lipoprotein transport influences how these hydrophobic molecules are distributed among tissues; *BCO2* can reduce the available precursor pool through asymmetric cleavage; *CYP2J19* and *BDH1L* convert selected yellow precursors into red ketocarotenoids; and feather-follicle deposition determines where pigments become visible. These steps are not independent. Reduced uptake can limit downstream colour production, high *BCO2* activity can deplete substrates before ketolation or deposition, and region-specific *CYP2J19* expression can convert a shared systemic carotenoid pool into local red ornaments. Carotenoid colouration is therefore better interpreted as the output of a connected uptake–cleavage–conversion–deposition network than as the consequence of a single gene acting in isolation.

### 2.3. Structural Colouration

Structural colouration differs from pigmentary colouration because hue is produced by light interacting with nanoscale feather architecture rather than by selective chemical absorption. This depends largely on spatial variation in refractive index at length scales comparable to visible wavelengths. Feather structural colours are produced by the physical organisation of keratin, air spaces and melanosomes, although their development remains controlled by cellular and genetic processes. Hummingbirds exhibit extraordinary structural colour diversity, mediated by specialised melanosome morphologies [111].

Non-iridescent blue and ultraviolet colours often arise from quasi-ordered keratin–air matrices within feather barbs. Air channels and keratin walls create refractive-index contrast, and coherent scattering reinforces some wavelengths while suppressing others. Blue structural colours in species such as blue jays (*Cyanocitta cristata*), eastern bluebirds (*Sialia sialis*) and budgerigars (*Melopsittacus undulatus*) arise from air–keratin nanostructures with channel diameters of roughly 100–200 nm and relatively uniform spacing [110]. Variation in air-channel diameter, keratin thickness, packing regularity and refractive-index contrast can therefore shift peak reflectance, saturation and brightness.

Iridescent colours usually involve a different structural organisation. They often arise from more ordered arrays in barbules, including layered keratin, melanosome arrays or combinations of melanin and keratin that act as thin films or multilayer reflectors. These structures generate angle-dependent colour because the optical path length changes with viewing geometry. Peacocks and hummingbirds provide examples in which ordered melanosomes and thin-film interference generate iridescent, angle-dependent colours. Melanosomes can therefore influence colour chemically, by absorbing light through melanin, and physically, by contributing to nanoscale optical architecture. Melanosomes therefore sit at the boundary between pigmentary and structural colour production.

The regional specification of feather nanostructures is under developmental genetic control. Genomic and developmental studies implicate signalling pathways such as BMP and WNT in barbule cell fate and feather morphogenesis [112,113,114]. These pathways may intersect with pigment-cell behaviour, although current evidence does not yet fully resolve whether nanostructure assembly and pigment deposition can be independently modified by mutation or selection. Such structural differences may also influence how non-iridescent and iridescent colours respond to developmental or genetic variation. Sex steroid hormones may further modulate ornament development, with effects that differ among melanin, carotenoid and structural colour systems.

### 2.4. Endocrine Links Between Colour-Production Mechanisms and Sexual Dichromatism

The pigment and structural pathways outlined above are embedded within endocrine and reproductive physiology. Sex steroid hormones can act as upstream modulators of colour expression, but the strength of gene-specific evidence differs among pathways. In carotenoid systems, androgens have been linked to the activity or expression of *CYP2J19* and *BCO2*, thereby connecting reproductive state with ketocarotenoid production, precursor retention and feather-region deposition [95,115]. In melanin systems, sex-biased regulation of *MC1R* and *ASIP* can alter eumelanin–pheomelanin balance [5,116]. Oestrogens and other endocrine signals may also influence moult timing, follicle physiology and ornament development, although their direct gene-specific roles in avian plumage colouration remain less well resolved. These endocrine effects provide mechanistic links between colour-production pathways and sexual dichromatism; their sex- and feather-region-specific deployment is discussed in Section 3.

## 3. Sexual Dichromatism in Avian Plumage

Sexual dichromatism—pronounced colour differences between males and females—is widespread across birds. It often reflects divergent selective pressures: conspicuous male ornaments may increase mating success, whereas more cryptic female plumage may reduce predation risk during nesting. At the molecular level, dichromatism usually results from sex-biased deployment of shared colour-producing pathways rather than the presence of entirely sex-specific pigment genes [116]. Endocrine regulation provides one upstream route through which these shared pathways may be deployed differently between sexes.

### 3.1. Genetic Mechanisms of Sexual Dichromatism

#### 3.1.1. Sex-Specific Gene Expression

Sex-specific *CYP2J19* expression provides one route to red sexual dichromatism [115]. In dichromatic species, male-biased *CYP2J19* expression can enhance ketocarotenoid production, whereas lower or absent expression in females limits red pigment deposition. Transcriptomic analysis of zebra finch cheek patches shows that region-specific sexual dichromatism is associated with spatially restricted *CYP2J19* expression and regulatory divergence in feather follicles [10]. Mandarin duck sail feathers provide a complementary developmental example of long-term sex-biased ornament regulation [117,118]; current evidence links this case to ornament development, not to a specific *CYP2J19*- or *MC1R*/*ASIP*-mediated pathway.

Sex-specific expression also has a temporal dimension. Some dichromatic phenotypes may arise during a narrow window of feather growth, whereas others depend on repeated endocrine input across moult cycles or long-term maintenance of ornamental feather structures. Transcript abundance measured in adult tissues may therefore miss the developmental window in which the colour phenotype was established. Future studies of dichromatism should sample actively growing follicles from defined feather tracts and developmental stages, not only mature feathers or whole-skin transcriptomes.

#### 3.1.2. Sexual Dichromatism and Gene Expression

Carotenoid-based plumage is frequently associated with sexual dichromatism in North American songbirds, consistent with visual signalling and sexual selection [60]. In mosaic canaries, males accumulate more carotenoids in feathers than females [115], indicating sex-biased regulation of a shared carotenoid-processing pathway rather than a wholly sex-specific pigment system. Androgen-associated *CYP2J19* expression may increase ketocarotenoid production in male feather follicles, whereas feather-region-specific *BCO2* expression may reduce the yellow precursor pool through cleavage. Thus, sex differences in carotenoid colouration may arise from differential precursor retention, enzymatic conversion and follicle-level deposition, although direct tests in growing sexually dichromatic feather tracts are still needed [115,119].

In the red siskin (*Spinus cucullatus*), sex-specific carotenoid pigmentation maps to a genomic region containing *BCO2* [115]. A plausible model is that sex-biased *BCO2* regulation changes carotenoid retention: lower activity in male pigmented feather tracts would allow more substrate to remain available for deposition, whereas higher activity in females would reduce colour intensity. Comparative cichlid studies support the broader principle that carotenoid-gene expression can be sex-biased in ornamented tissues [119], although the avian mechanism requires direct testing.

#### 3.1.3. Polygenic Control

Genetic control of sexual dichromatism is often polygenic [120]. In the American goldfinch, a complex colour phenotype involves multiple loci, including genes associated with carotenoid absorption, transport and deposition [87,120]. Parrot colouration is also controlled by a network of genes underlying psittacofulvin biosynthesis and modification [107,108]. Thus, major loci such as *CYP2J19* can have strong effects, but the visible phenotype usually emerges from networks that connect pigment chemistry, transport, feather-region expression and developmental timing.

## 4. Biochemistry and Physiology of Carotenoid Colouration

### 4.1. Carotenoid–Protein Interactions

Carotenoid-based colours are affected not only by the pigment concentration but also by the interaction between carotenoids and feather keratins [121]. These interactions modulate optical properties, generating hue variation from yellow to red through hydrophobic associations and potential covalent binding [122].

Comparative analyses of manakins (*Pipridae*) show that identical carotenoid molecules can generate different colour phenotypes through protein-binding context [123]. Broader passerine analyses likewise indicate that metabolic transformation and pigment–protein interaction jointly shape plumage colour diversity [92,123]. However, the feather proteins responsible for these colour-determining interactions remain poorly characterised. Quantitative proteomics of feather barbule extracts, integrated with transcriptomic and metabolomic data, would help identify the binding proteins and structural features that tune carotenoid hue [121,122,123].

### 4.2. Physiological Costs and Trade-Offs

Having described carotenoid uptake, processing and deposition, this section considers why carotenoid traits are especially sensitive to physiological state and environmental variation. Carotenoid colouration is a condition-dependent trait linked to nutrition, metabolism and health. The Hamilton–Zuk hypothesis proposed that elaborate ornaments can act as honest signals of parasite resistance [124], shifting attention from pigment identity to the selective pressures maintaining costly display. In carotenoid-based plumage, these costs extend beyond a simple trade-off between ornamentation and immunity. Because carotenoids are acquired from food and carotenoid-derived compounds can contribute to antioxidant buffering, immune modulation, visual physiology, epithelial maintenance and lipid-associated metabolism, allocation to plumage may compete with other somatic demands. During moult, pigment allocation also occurs alongside feather growth, keratinisation and the formation of structurally functional ornaments. Experiments on yellow warblers and studies on captive house finches support the view that diet, parasite load, mitochondrial function and carotenoid metabolism interact to shape colour expression [12,125,126].

A bright carotenoid-based signal may therefore reflect not only pigment abundance, but also the ability to maintain physiological function while routing sufficient carotenoid precursors through uptake, transport, conversion and deposition pathways. A dull colour can arise for several different reasons. Reduced ornament expression may reflect low dietary supply, inefficient SCARB1/CD36-mediated uptake, altered lipoprotein transport, lower hepatic conversion, stronger *BCO2*-mediated cleavage, weaker *CYP2J19*/*BDH1L* activity, or reduced follicle deposition. These alternatives carry different ecological meanings: some indicate environmental limitation, others indicate physiological allocation, and still others reflect lineage-specific metabolic architecture. The reliability of carotenoid-based signals can therefore be tested more directly by measuring pigment availability, expression of pathway genes, physiological state and reproductive or survival outcomes in the same ecological context.

### 4.3. Species-Specific Patterns

Distinct avian lineages exhibit divergent capacities for carotenoid accumulation. In the white stork (*Ciconia ciconia*), astaxanthin predominates in plumage, correlating with dietary intake of invasive crayfish (*Procambarus clarkii*) [73]. Similarly, carotenoid deposition in the great frigatebird (*Fregata minor*) throat pouch reflects species-specific metabolic capacities [127]. Comparative analyses of the Icteridae reveal that carotenoid feather composition tracks phylogenetic relationships [128], suggesting conserved metabolic pathways within clades.

Species-specific patterns also caution against overgeneralising from a single model organism. A dietary carotenoid that produces intense ornamentation in one lineage may be stored, metabolised or degraded differently in another. For example, the white stork case links pigment expression to ecological diet, whereas the frigatebird throat pouch illustrates tissue-specific deposition outside feathers. Icterid comparisons further show that phylogenetic history can shape which carotenoids are retained or transformed. These examples support a comparative framework in which diet, physiology and ancestry are treated as interacting variables rather than competing explanations.

### 4.4. Evolutionary Variation in Carotenoid Assimilation

Birds show substantial interspecific variation in carotenoid assimilation efficiency [60]. At macroevolutionary scales, ecology, life history and sexual selection jointly predict colour strategies across the avian tree of life [129], while photobleaching shows that abiotic weathering can also shape plumage phenotype expression [130]. Environmental availability constrains carotenoid expression, but species-specific metabolic capacity determines how available pigments are absorbed, transported and deposited [87]. Because carotenoids are lipophilic, this variation is mechanistically tied to lipid metabolism and lipoprotein transport. These physiological differences provide the raw material on which selection can act, linking molecular mechanisms to evolutionary patterns.

## 5. Evolutionary Perspectives

Convergent plumage colour can be interpreted at several mechanistic levels. Red carotenoid plumage provides one set of examples. In cotingas, ketocarotenoid-based red colouration involves modification of the *CYP2J19* pathway [131], whereas recurrent red colouration in weaverbirds is associated with *CYP2J19* gene-family expansion and functional divergence. Hybrid-zone studies further show that *CYP2J19* allelic variation can track plumage phenotype across population boundaries [28,95,120]. These cases illustrate convergence through repeated use, modification or diversification of the same broader carotenoid-conversion pathway.

Other red phenotypes show that similar colours need not always arise through the same biochemical route. Population-scale work has shown that convergent colouration may be underpinned by shared haplotypic modules at a single gene [9], while supergene architecture and adaptive introgression can link colour to morphology or spread carotenoid alleles across geographic gradients [132,133]. House finches provide a contrasting case, discussed above, in which red plumage can arise without detectable reliance on the canonical *CYP2J19* route. Together, these examples indicate that routes to red are multiple but constrained by enzymatic feasibility, phylogenetic history and the biochemical boundaries of carotenoid metabolism [134].

Depigmentation provides a parallel set of cases in the melanin system. As outlined above, white plumage can result from *EDNRB2*-dependent failure of melanocyte colonisation, *RAI14*-associated retinoid-signalling disruption, *MITF* structural variation or disruption of pigment-synthesis enzymes such as tyrosinase. These examples show that convergent depigmentation can arise through disruption of different levels of the pigmentation hierarchy, from cell migration and differentiation to transcriptional architecture and pigment synthesis. These cases shift the evolutionary question from whether colour phenotypes converge to which mechanistic level is reused or modified.

Feather morphogenesis provides the developmental context in which pigment deposition evolves [114]. Region-specific BMP, WNT and SHH signalling gradients specify distinct feather types across body tracts [16,135,136], while activator-inhibitor logic can generate periodic colour patterning within individual feathers [137]. A conserved molecular template underlying colour pattern diversity in estrildid finches further suggests that phenotypic diversity often results from quantitative modulation of shared developmental programmes rather than from de novo pathway innovation [138].

From a deep-time perspective, the stepwise elaboration of feathers in non-avian theropods likely created new spatial contexts for pigment deposition [139,140]. Selection therefore acts on integrated colour-structure phenotypes: pigment chemistry, feather architecture and avian visual perception jointly determine which signals are produced, detectable and favoured. For this reason, evolutionary interpretations of plumage colour need to track both the pigment route and the developmental substrate in which it is expressed.

These mechanisms also explain why pigment systems are rarely isolated within the organism. Carotenoid availability may modulate melanin synthesis, and vice versa, creating condition-dependent trade-offs in pigment allocation [95]. Such biochemical interactions expand the achievable colour space and may facilitate the evolution of novel ornamental phenotypes. Comparative genomic and transcriptomic analyses increasingly show that colour pattern evolution across vertebrates often proceeds through changes in the spatial and temporal deployment of conserved pigmentation gene networks [6,7]. This regulatory logic links pigment chemistry, developmental patterning and lineage-specific colour diversification.

## 6. Future Research Directions

The synthesis in Section 2, Section 3, Section 4 and Section 5 points to four methodological priorities. First, regulatory causality needs to be tested by combining comparative transcriptomics of matched growing feather tracts with chromatin-accessibility assays, enhancer-reporter tests and, where feasible, CRISPR/Cas-based perturbation in tractable avian cell, embryo or feather-follicle systems. Second, biochemical flux needs to be measured more directly using metabolomics, pigment chemistry and stable-isotope tracing across moult stages. Third, structural colour requires the integration of developmental perturbation with quantitative optics, electron microscopy and avian visual modelling. Fourth, ecological validation requires field studies that measure diet, physiological state, colour output, mate choice or survival in the same individuals. Together, these approaches would help connect candidate genes to causal mechanisms, developmental processes and ecological outcomes in plumage colour evolution.

### 6.1. Regulatory Mechanisms

The sex-specific and tissue-specific expression of the key pigmentation genes CYP2J19 and BCO2 remains incompletely characterised. Identification of upstream transcription factors, cis-regulatory elements, and epigenetic modifications (DNA methylation and histone acetylation) governing these expression patterns will clarify how localised colour phenotypes develop.

Regulatory work should move from expression–phenotype correlations towards tests of causality. Promoter and enhancer regions near *CYP2J19*, *BCO2*, SCARB1 and *ASIP* are obvious starting points, but the critical sampling unit is the growing feather follicle rather than the whole skin or liver alone. Reporter assays, chromatin accessibility data and targeted perturbations, where feasible, would help determine whether candidate variants are sufficient to alter pigment deposition in a specific feather tract.

### 6.2. Alternative Routes

The molecular basis of red colouration in *CYP2J19*-independent lineages (e.g., house finches, *Haemorhous mexicanus*) represents a critical unresolved question. Whether alternative cytochrome P450 enzymes or entirely distinct enzymatic routes (e.g., non-enzymatic oxidation) facilitate ketocarotenoid production requires functional characterisation.

The immediate need is biochemical rather than purely comparative: candidate enzymes must be tested against relevant carotenoid substrates, and negative results are also informative because they delimit the feasible routes to red plumage.

### 6.3. Epigenetics

Epigenetic work should be framed cautiously. Diet or stress may alter methylation or chromatin accessibility around loci such as *BCO2* or SCARB1, but demonstrating adaptive plasticity requires showing that such marks change expression during feather growth and, where possible, persist long enough to affect later moult or reproductive episodes.

### 6.4. Functional Trade-Offs

Carotenoid allocation to plumage competes with immune function, antioxidant defence and visual pigment maintenance. The strongest tests of honest signalling will therefore not be simple colour measurements but manipulations that connect pigment supply, physiological state, ornament expression and reproductive success in the same individuals.

### 6.5. Evolutionary Dynamics

Connecting genotypes to adaptive phenotypes across ecological and phylogenetic scales requires integrating population genomics with field-based studies of selection. Comparative analyses across replicate lineages will elucidate the relative contributions of sexual selection, natural selection, and genetic drift to colour allele frequency evolution. Advances in visual ecology—including modelling of receiver perception, quantification of colour distances in avian tetrachromatic colour space, and linkage of signal properties to habitat-specific visual backgrounds—are transforming this integration by grounding genotype–phenotype connections in an ecologically realistic perceptual framework [23,25]. Expanding genomic resources for domesticated and wild birds is simultaneously accelerating the identification of causative variants underlying colour polymorphisms [59], while integrative reviews continue to frame the open questions about pattern formation, honest signalling, and macroevolutionary diversification that population genomic approaches must ultimately address [7,8].

Automated phenotyping will also matter. Quantitative image-analysis approaches can measure patch shape, texture and multidimensional colour variation more consistently than manual scoring, but these measurements should be combined with avian visual modelling and standardised imaging conditions because avian receivers may discriminate colour dimensions that are not apparent to humans [141]. Their value will be greatest when automated traits are analysed together with genomic data, rather than treated as descriptive image features alone.

### 6.6. Structural–Pigmentary Interactions

A central unresolved question is whether the developmental programmes governing nanostructure assembly and pigment deposition can be modified independently by mutation or selection. BMP, WNT and Agouti signalling nodes have been implicated in feather morphogenesis, nanostructure assembly and pigment-cell behaviour [114]. The key issue is separability: do these regulators control structural and pigmentary outputs through distinct downstream branches, or are the two outputs necessarily co-regulated? Experimental perturbation of follicle development, combined with quantitative optics, will be needed to answer this question.

### 6.7. Environmental Plasticity

Environmental plasticity provides a direct test of how genetic constraints on pigmentation interact with ecological change. The conservation-relevant issue is which part of the pathway makes a signal vulnerable when carotenoid ornaments fade under poor conditions. Species with narrow dietary carotenoid sources or limited enzymatic flexibility in pathways involving *CYP2J19*, *BCO2* and SCARB1 may be more sensitive to reduced carotenoid access, but this remains a prediction. Field studies that combine diet surveys, pigment chemistry, gene expression and visual modelling across environmental gradients will be needed to test it.

### 6.8. Multi-Omics Integration

No single molecular layer can fully resolve the mechanistic questions reviewed here. Transcriptomics identifies expressed genes but cannot determine why pigmentation genes are activated or silenced in specific sexes, tissues or feather regions. Epigenomics can reveal chromatin accessibility, DNA methylation and histone modifications that regulate these expression patterns; proteomics can identify pigment-binding proteins and post-translational changes; and metabolomics can quantify carotenoid precursors, ketocarotenoids and melanin intermediates. Integrating these layers in the same growing feather follicles would link regulation, biochemical flux and visible colour output.

Multi-omics studies will be most useful when they are comparative and time-resolved. In carotenoid traits, precursor availability, transport and conversion gene expression, pigment-binding proteins and regulatory accessibility need to be measured across the same moult trajectory. In melanin and structural traits, separating melanocytes, keratinocytes and follicle niche cells will help distinguish differences in pigment-cell number, enzyme expression, nanostructure assembly and regional tissue patterning. The goal is not to generate larger gene lists but to connect molecular layers to the visible colour produced by a growing feather.

## 7. Conclusions and Perspectives

Avian plumage colour diversity arises from interactions among colour-producing mechanisms, feather-region-specific regulation and the visual and ecological contexts in which signals are expressed. At the mechanistic level, melanin, carotenoid, psittacofulvin and structural colour systems generate visible colour through different biochemical or optical routes. At the regulatory level, these systems are deployed unevenly across sex, tissue and feather regions. At the ecological level, diet, habitat and receiver vision influence which signals are expressed, maintained and selected. These interactions explain why similar plumage colours can arise by changing pigment chemistry, altering feather-region expression, modifying the feather architecture or combining these processes in different lineages.

This synthesis supports four main conclusions. Melanin and carotenoid pigmentation systems operate through distinct but partly convergent genetic pathways, with key genes such as *MC1R*, *ASIP*, *CYP2J19*, *BCO2* and SCARB1 serving as major determinants of colour variation. Colour evolution often depends on regulatory deployment, including sex-specific, tissue-specific and feather-region-specific expression, as much as on coding changes. Similar colour phenotypes, especially red carotenoid plumage, can evolve through both canonical *CYP2J19*-mediated routes and alternative mechanisms. Finally, feather nanostructure and pigment deposition share developmental contexts, so colour-pattern evolution must be interpreted within feather morphogenesis as well as pigment chemistry.

The figure separates proximate colour-producing mechanisms from evolutionary and ecological outcomes. Androgens/testosterone are shown as upstream modulators that may influence melanin, carotenoid and structural colour systems to different degrees.

(1) Melanin system: α-MSH activation of *MC1R* promotes *MITF*-mediated expression of *TYR*, *TYRP1* and *DCT* and favours eumelanin production, whereas *ASIP* antagonises *MC1R* and shifts output towards pheomelanin. The golden pheasant (*Chrysolophus pictus*) illustrates *ASIP* alternative splicing within this axis. *EDNRB2* is shown separately because it controls melanocyte migration and can produce white or piebald plumage through distribution failure.
(2) Carotenoid and psittacofulvin systems: Dietary carotenoids are absorbed through SCARB1/CD36, transported by lipoproteins and either deposited directly as yellow pigments or converted to red ketocarotenoids through the canonical *CYP2J19*–*BDH1L* route. *BCO2* limits the available yellow pool through asymmetric cleavage. House finches represent a *CYP2J19*-independent red route whose precise mechanism remains unresolved. Parrots are included separately because psittacofulvins arise from an endogenous *MuPKS*/*ALDH3A2* pathway rather than from dietary carotenoids.
(3) Structural colouration: A blue colour arises from coherent scattering by air–keratin nanostructures, whereas iridescence in peacocks and hummingbirds reflects ordered melanosomes and thin-film interference. Developmental regulators such as BMP and WNT may influence both nanostructure assembly and pigment deposition, but their separability remains an open question.
(4) Sexual dichromatism: The region-specific expression of genes such as *CYP2J19* and *BCO2* provides a mechanism by which the same pigment-processing pathway can be deployed differently between sexes and feather regions.
(5) Evolutionary outcomes: Repeated red plumage, sexual dichromatism, signal reliability and environmental sensitivity reflect lineage-specific changes in pigment chemistry, gene regulation and feather development. The Hamilton–Zuk hypothesis links carotenoid ornament expression to condition-dependent signalling.

Solid arrows indicate established flow; dashed arrows indicate regulatory influence or crosstalk; and open questions indicate unresolved molecular mechanisms.

This review connects older descriptive work on plumage colour with biochemical pathways and genomic evidence. *CYP2J19*-independent red colouration illustrates why this integration is necessary: even apparently well-characterised colour systems can contain alternative biochemical solutions. Resolving such cases will require transcriptomic, proteomic and metabolomic approaches applied within the same experimental systems, together with ecological measurements that connect genotype, molecular phenotype and organismal fitness.

Convergent depigmentation provides a clear example of modularity. White plumage can arise through *EDNRB2*-dependent failure of melanocyte colonisation, *MITF* structural variation, *RAI14*-associated retinoid-signalling disruption or loss of pigment-synthesis enzymes such as tyrosinase. A single visible phenotype can therefore result from disruptions at different levels of the pigmentation hierarchy. Comparative structural-variant profiling and single-cell transcriptomics of feather follicles would help estimate how often each route has evolved across birds.

These links may also be useful for conservation-oriented studies. Signal expression can depend on genetic capacity, dietary carotenoid availability and the metabolic costs of ornamentation, all of which may vary across environments. Long-term studies that combine colouration genetics, pigment chemistry and ecological monitoring could help test when plumage traits provide reliable indicators of environmental change.

## Data Availability

This review article synthesises published research findings. No new experimental datasets were generated or analysed for this study. All cited studies are available through their respective publications listed in the references.
